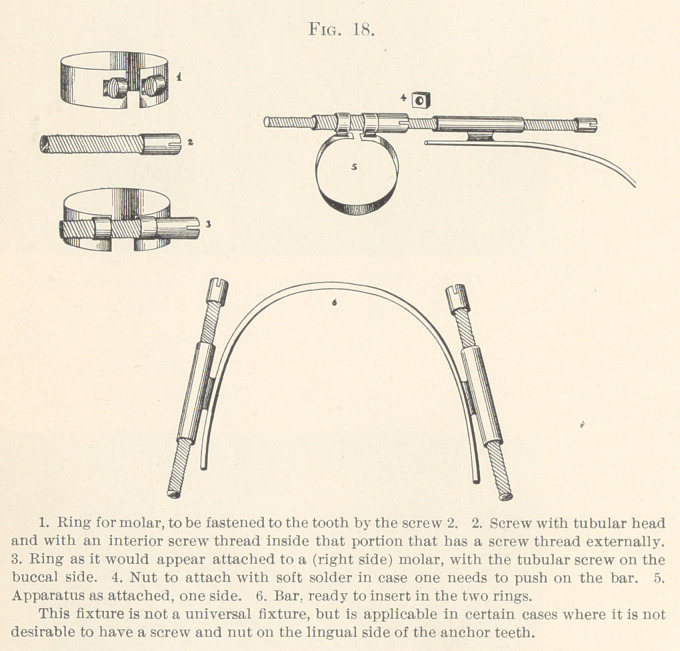# Some of the Causes of Irregular Teeth, with Suggestions as to Preventive Treatment or Early Cure

**Published:** 1903-04

**Authors:** E. A. Bogue


### International Dental Journal.

Vol. XXIV. April, 1903. No. 4.

### Original Communications.[1]

### SOME OF THE CAUSES OF IRREGULAR TEETH, WITH SUGGESTIONS AS TO PREVENTIVE TREATMENT OR EARLY CURE.[2]

BY E. A. BOGUE, M.D., D.D.S.

Since dentistry has been recognized in this country efforts have been made to correct irregularities in the positions of the teeth.

Some of these efforts have been successful, some have been failures; but why success has been attained in one case and failure has resulted in another does not seem to have been clearly explained in any of the treatises thus far written on the subject.

It is necessary therefore that we should look into the causes of the irregularities, before we can understand the principles that underlie their successful cure.

I use the word *cure* in its original sense,—care, direction, management.

---

[1] The editor and publishers are not responsible for the views of authors of papers published in this department, nor for any claim to novelty, or otherwise, that may be made by them. No papers will be received for this department that have appeared in any other journal published in the country.

[2] Read before The New York Institute of Stomatology, March 3, 1903.

It must be remembered that we are formed in two halves, and that those two halves unite to form the perfect creature.

During the processes of development, a very slight cause may produce as effect a wide deviation from the typal form.

For example, the pressure of the lower jaw of an infant "in utero" is sufficient if it closes beneath and into the upper jaw to form a cleft in the hard palate and to widely separate the two halves of the superior maxillary.

The proof of this is, according to Dr. Brophy, that when the separated halves are brought together by his operation for cleft palate, the upper jaw is found to be in proper relation to the lower, and the eruption of the teeth takes place nearly along normal lines.

When the question, What are normal lines? is presented, we are confronted with Dr. Cryer's conclusion in his recent work,—that "the idea of an unvarying typal form in anatomy is not correct." That if a typal bone could be found or devised and a thousand real ones examined, it is possible, though doubtful, that in the thousand bones two or three could be found which exactly corresponded with the typal bone.

When, therefore, we undertake to study the human teeth and to understand what are normal teeth, in their normal positions and relations each to the other, we have to examine large numbers of teeth, or models of them, that are presumably as accurate in their formation and arrangement as can be found.

I shall present now but one specimen, that represents, as far as may be, a perfect denture.

The main characteristics of the so-called normal denture are, however, all there, as far as they are required for our purpose this evening.

The main curves, which have to do with the application of the greatest degree of force in mastication without injury to these organs, are there. The inclination towards the tongue of the lower molars, in order to properly cusp and antagonize with the upper molars, which are implanted in a jaw smaller than the lower, is there as well, and also the outward incline of these upper molars.

The curves from before backward are distinctly shown, and all these curves are of practical utility considering the form and construction of the teeth.

Blainville (Owen, page 515), in his "Osteography of Vertebrate Animals," calls the bicuspids the "pre-molars," the first permanent molar the principal molar, and the other two teeth back of this he calls post-molars.

He next points out that "another characteristic in the principal molar of man is its position, being implanted below the root of the zygomatic process of the upper jaw."

It is so largely with this *principal molar* that we have to do this evening, that I beg to draw your attention carefully to it.

As the lower jaw develops first, and usually the lower teeth also, we will begin by noticing that the normal lower first molar has upon its outer or buccal side three cusps or tuberosities. Upon the inner or lingual side it has two.

The upper molar has two on either side. But in the normal position of these teeth, when closed, the anterior buccal cusp of the upper molar and the anterior lingual cusp sit astride of the outer ridge of the lower first molar just posterior to, and nicely fitting in with, the anterior buccal cusp of the lower molar.

When at their eruption these teeth come into just this position on both sides of the mouth, we may be certain that there will be no serious irregularity back of the cuspid teeth. But when from any cause these two principal molars erupt out of line with each other, —that is to say, with the upper molar too far forward of the position just described, or too far backward, or too far on the lingual or buccal side for the two teeth to come accurately into the position described,—irregularity of some sort is absolutely certain.

The reason for this is not very far to seek.

The teeth of each individual are formed of a size and type adapted to that individual.

There is just room enough in each skull for the typal jaw belonging to that skull to develop, and to erupt the typal teeth for that jaw.

But if, during infancy or early childhood, some slight cause for a deviation obtrudes itself, and continues for a little time to act, with only as much force as might be exerted by a single hair, there is sure to be a deviation from the typal form, or position, or both.

If now either of the principal molars (which, antagonizing each other, stand exactly at the middle, and bottom, of the curve extending from the top of the lower cuspids to the top of the wisdom-tooth below) is thrown out of position, the remainder of the teeth, anterior to the dislocated one, will not have the room in which to erupt.

For example, suppose the upper molar occludes with the lower, a little in advance of its proper position.

There must be room between the anterior surface of the first permanent molar and the spot where the cuspid should come down for the two bicuspids.

If there is not room, the forward pressure of the erupting molar will crowd the bicuspids in some direction, inevitably; either forward, making protrusion of the anterior part of the jaw, or laterally, towards the tongue or the cheek, breaking the arch and exhibiting irregular bicuspids which do not cusp.

The same condition of things is true if, by chance, the *lower* first molar erupts out of position.

To you who have studied occlusion I hardly need to say that I regard the replacement and retention of the first permanent molars into their proper and normal positions in relation to each other, should they be found developing out of place, at five and a half to seven years of age, just as essential as the replacement of the head of the femur into the acetabulum, before five years of age, should it be found dislocated.

What difference is there between a dislocated femur and a dislocated tooth? Both lead to serious deformity. The one involves limping on the pathway of life, being a cripple; the other involves limping through one's victuals, being a dental cripple; the proper function of mastication is not fulfilled; the results no one can foresee, but we know they are evil; always ugly, sometimes repulsive; sometimes fatal.

Most of these deformities may be at this tender age far more easily corrected than the dislocated hip. It may need a hospital where the patient is kept a month or two; sometimes it can be done in a few days, without disturbing, for more than a few minutes at a time, the usual avocations of the child.

But it can be done, and if done during this plastic age it is done painlessly, quickly, and, if under the eye of one who comprehends the situation and who understands "the total depravity of inanimate things" (children included), the teeth can be guided into place and held there.

This gives opportunity for development to go on in the centre of the region occupied by the two jaws; for additions to be made to the skull, to the brain occupying it, to the face, which needs the added dignity given by growth, or, as we say, by years, and it needs that that growth shall take place in a region out of sight, but closely related to those parts that are *in sight,* and upon which depend the impressions that our fellow-men are to get of us through the sense of sight.

These cranial additions take place mostly between the first permanent molar and the wisdom-tooth, and its surroundings. Very little, comparatively, of these additions take place in the anterior portions of the jaws, but what does take place there mostly occurs between the points of the cuspid teeth, and in the area formed by the incisors that *should* arch between them, but sometimes do not, themselves being jumbled by an extension of that irregularity of proceeding which began with the first permanent molar.

Those who have extracted the first molar, or, indeed, any other members from the arches, can never know what they have abstracted from those regions lying contiguous to the seat of intelligence and the centre of expression. The weak and flattened faces seen on all sides are witnesses to the loss.

I will show you this evening models taken a year ago of a child six years of age, where the temporary teeth were nearly regular and the incoming first permanent molars were seemingly most regular.

I will show you also models of the same child taken ten months later, which show the first permanent upper molars an entire cusp in advance of the corresponding lower molars, which shows the two upper central incisors projecting three-eighths of an inch beyond the tips of the lower central incisors just erupting; these upper centrals forming an obtuse V-shape, and almost preventing the closure of the lips over them. The lower centrals, in their half-erupted state, touching the upper gums, just posterior to the point at which the greatest tuberosity of the upper incisors still lies beneath the gum.

What has caused this great change in ten months? Not thumb-sucking; she does not do it. Not sucking a bottle; she was weaned years ago. I'll tell you what: adenoids no doubt obstruct the air passages; the child is a mouth-breather, though her mother did not know it until I called attention to it. This habit of keeping the mouth open has drawn upon the levator muscles almost constantly, resulting in a slight flattening of the upper arch in the region of the temporary cuspids.

This, by drawing the ends of the arch closer together, has thrown out the middle of the arch, giving the obtuse V-shape spoken of.

But that is not all; the child occasionally closed the teeth, and, in closing, the lower cuspids struck this narrowed upper arch in a way that was disagreeable, perhaps even slightly painful.

To get relief she closed the teeth a very little to one side, probably the left, where she obtained rest upon the incoming permanent molars above and below and upon the outer cusps of the temporary molars.

This seeking for repose became habitual, to such an extent that when the permanent molars erupted upon the other side of the mouth, they were not only out of line with each other, but they did not cusp,—that is, the long cusps did not fit into the depressions of the antagonist tooth.

It is this cusping, or fitting in of cusps to corresponding depressions, that keep the teeth in position.

The eruptive force of the upper molar, seeking to enlarge that region before mentioned in the interior of the head, has pressed forward the temporary molars and cuspids, and has thus itself gone farther forward than it should, having been left free to move by the teeth closing upon the ends of the cusps on the other side of the mouth when they closed there to get rest.

After the eruption and pressing forward of the left upper molar, the place of rest was apparently changed to that side, and the same process just described has taken place on the right side.

Result: A most serious deformity impending, which has come entirely within the last ten months, and which, if left alone, will, when adolescence or adult life is reached, take years to correct.

Why should it not be done now?

The influence of occlusion, which means a proper meshing of the cusps of the teeth into the depressions of their antagonizing teeth in the opposite jaw, has not been sufficiently taken into account in efforts at regulation.

That is one reason, and a very potent one, why, as mentioned in the beginning of my paper, some regulating cases have been successful and some have failed; and no one seemed to know the reason for either condition.

Without this cusping there is literally nothing except the tongue and lips to keep the teeth accurately in their places; and inasmuch as each molar tooth normally occludes with two other teeth, and is itself held from rotation by these two other teeth, we know of no force save the tongue and lips that helps to maintain the arches.

The accuracy of this assertion may be tested by making exact models of regular sets of thirty-two teeth and hinging them, so that they open horizontally from front backward, showing the contact of the occlusal ends of all the teeth from the lingual side.

A curious instance of the tendency of teeth to return in position to the typal form is shown in the case of Dr. Baker's son, drawings of whose teeth were exhibited before this body at the January meeting.

Having asserted that the young man's teeth, when freed from the restraint of retaining plates, would undergo very considerable changes, I asked Dr. Baker to send me models of the teeth as they now are. He kindly complied and I now have models taken in 1892, 1898, and 1903.

The oval form of the tongue is asserting itself in the rearrangement of the arches, which are being elongated from before backward and consequently flattened at the sides. The intermeshing of the cusps is becoming very much more accurate than it was when under the restraint of the retaining plates.

## Figures and Tables

**Figure f1:**
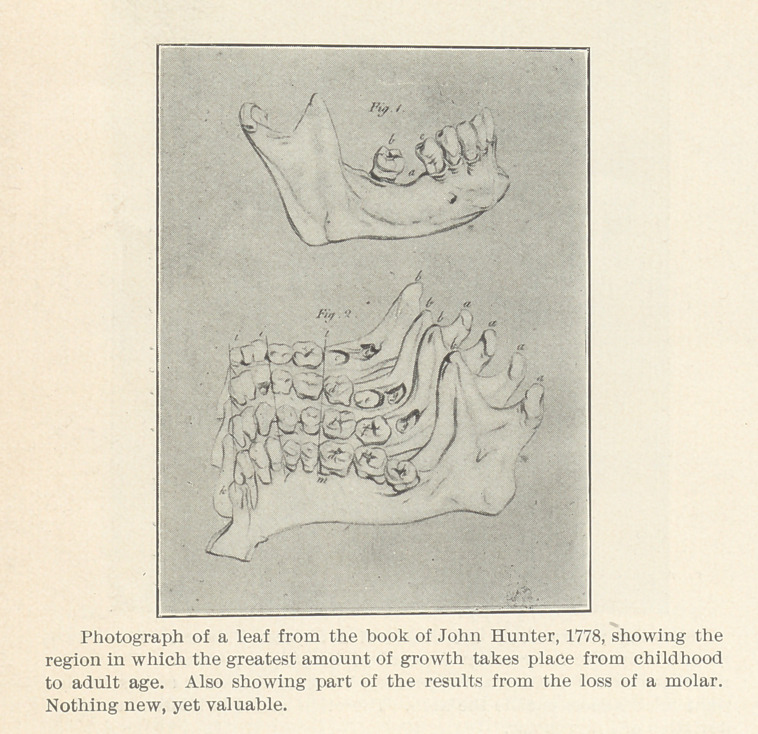


**Fig. 1. f2:**
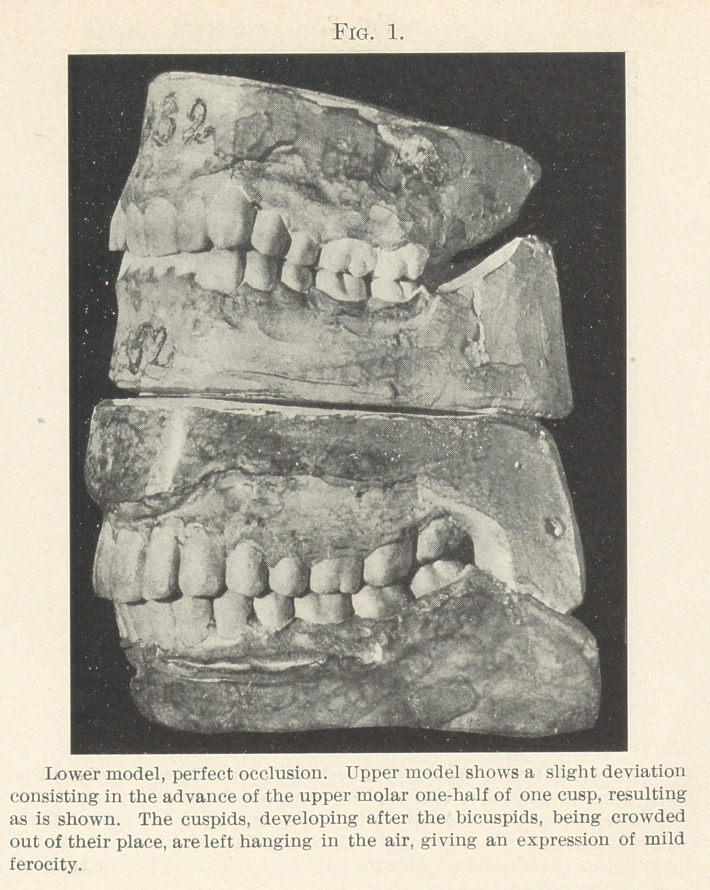


**Fig. 2. f3:**
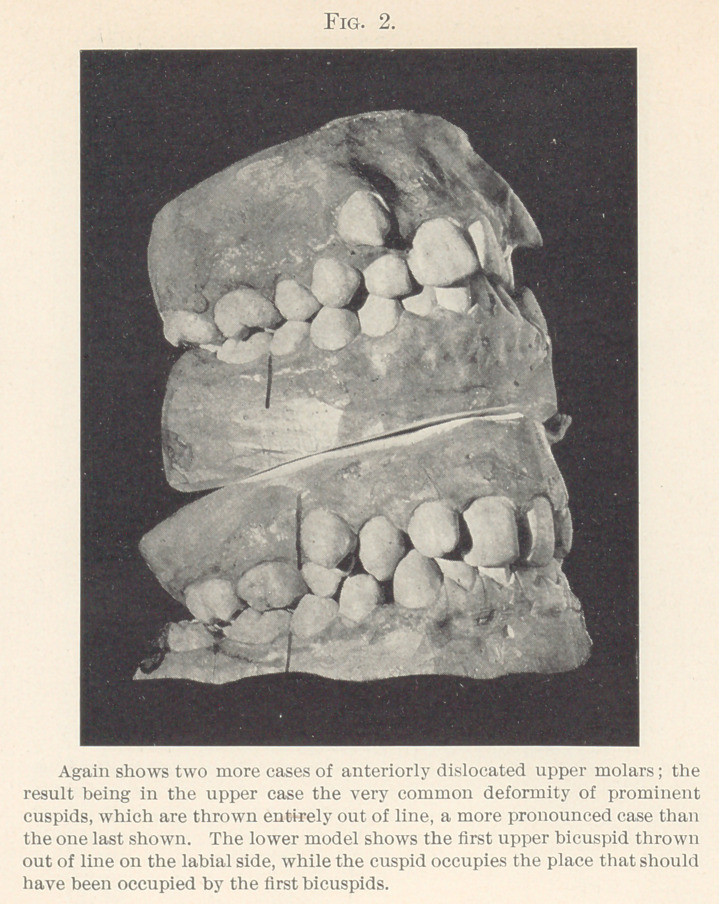


**Fig. 3. f4:**
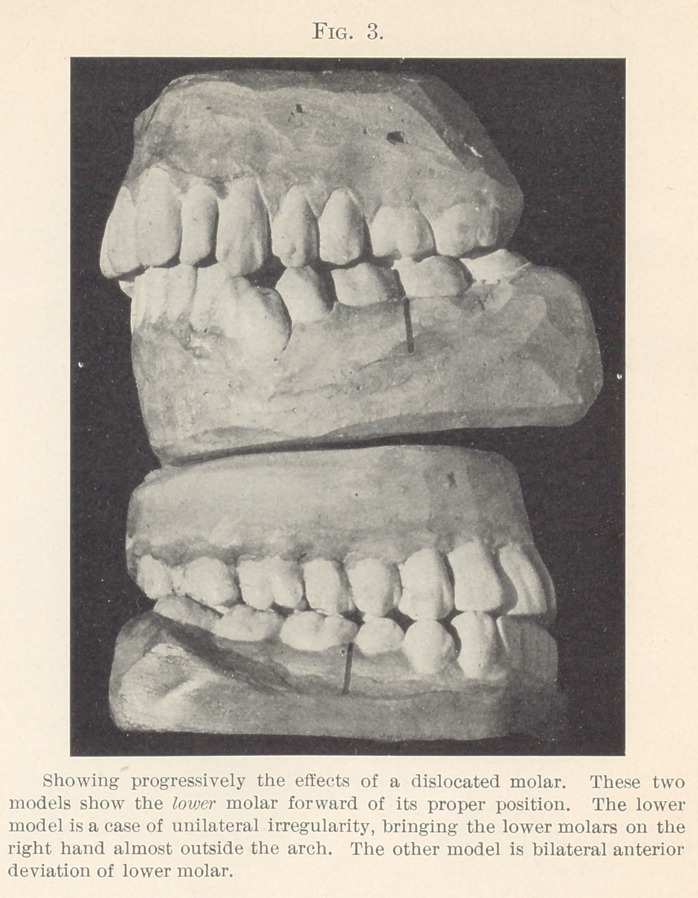


**Fig. 4. f5:**
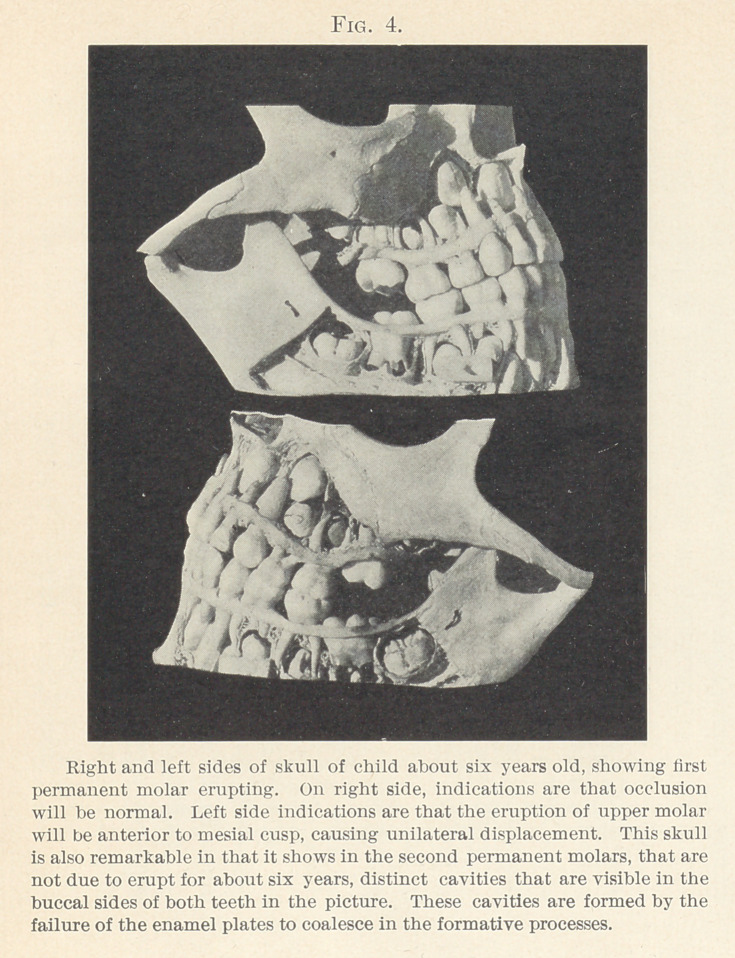


**Fig. 5. f6:**
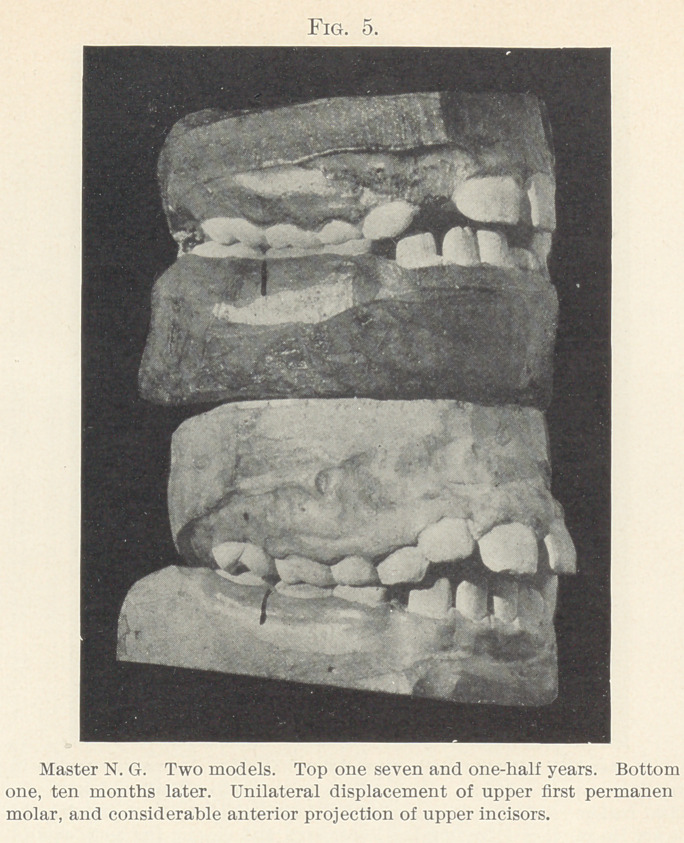


**Fig. 6. f7:**
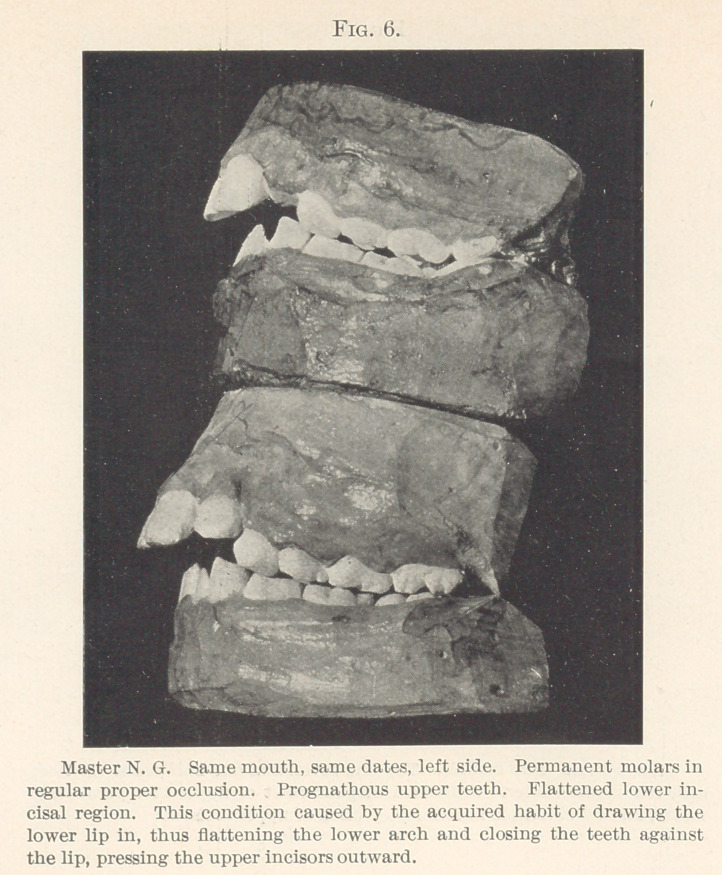


**Fig. 7. f8:**
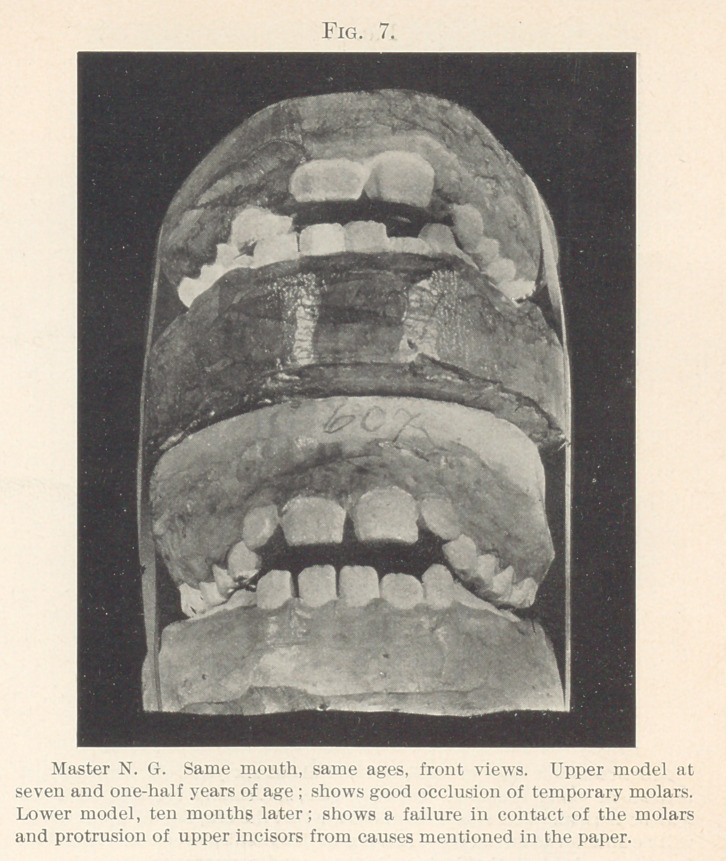


**Fig. 8. f9:**
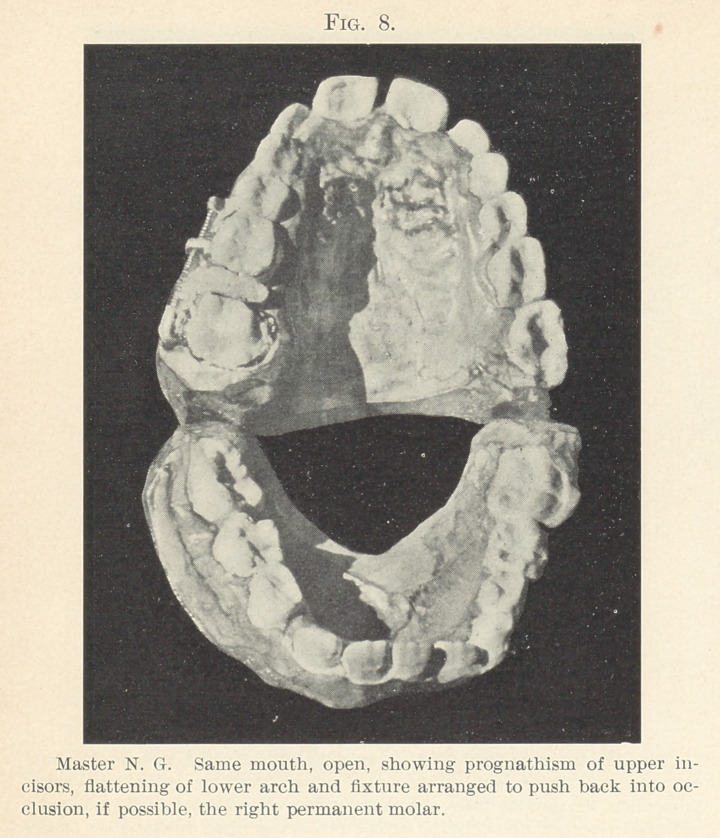


**Fig. 9. f10:**
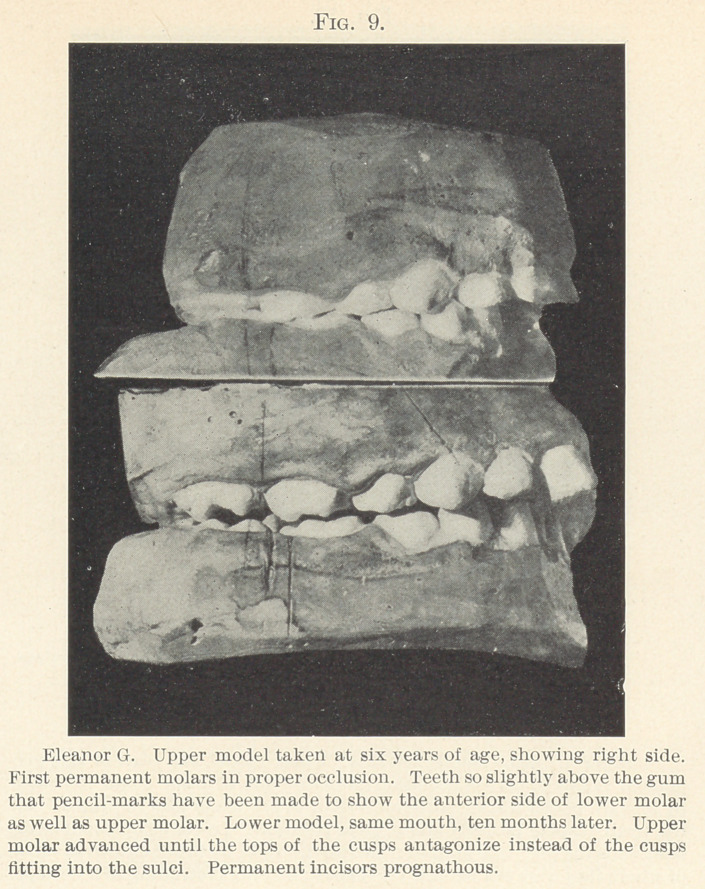


**Fig. 10. f11:**
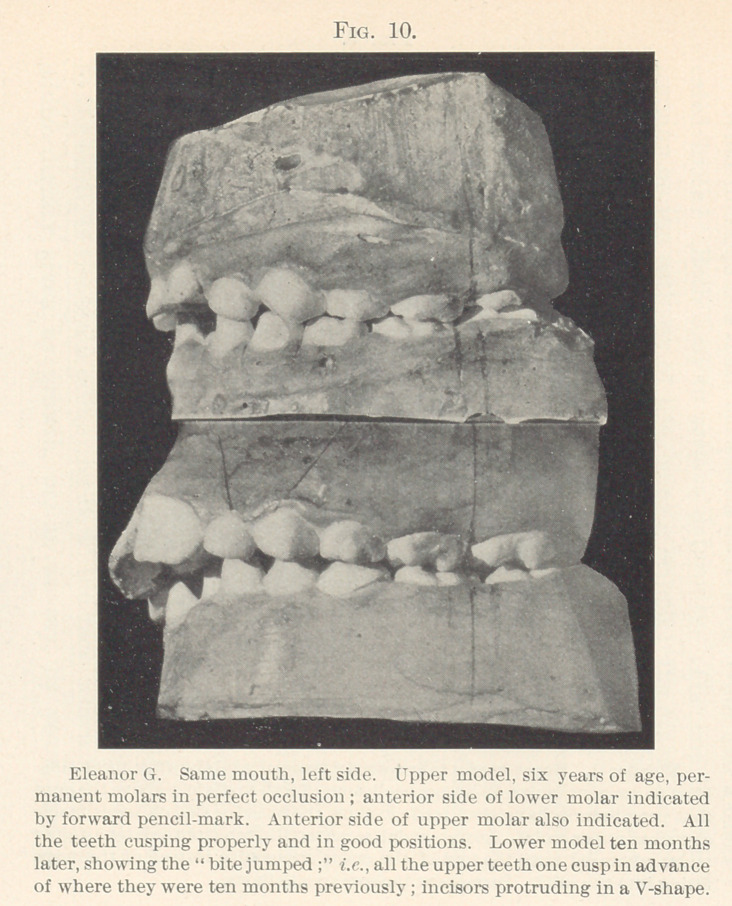


**Fig. 11. f12:**
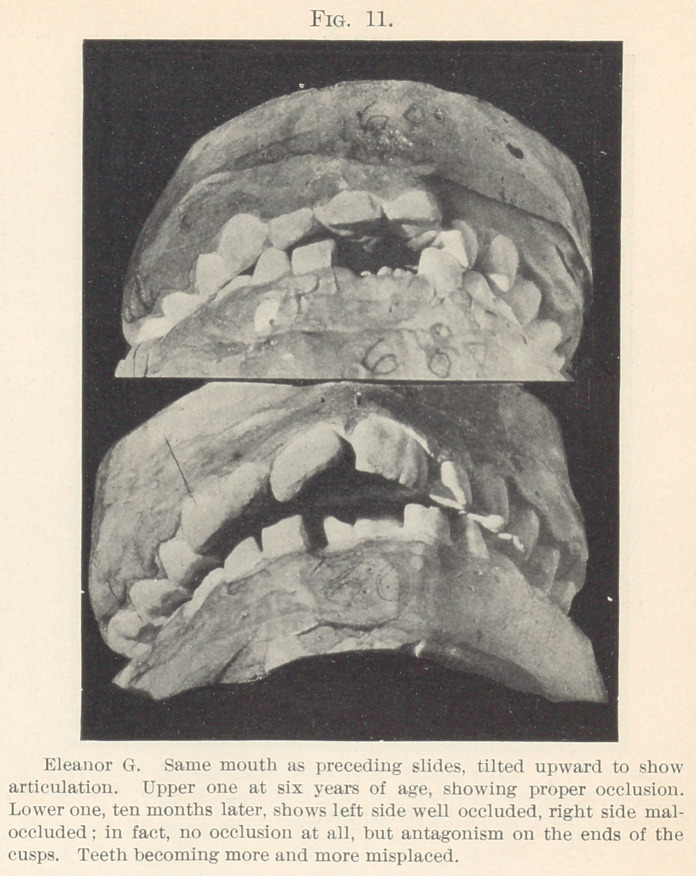


**Fig. 12. f13:**
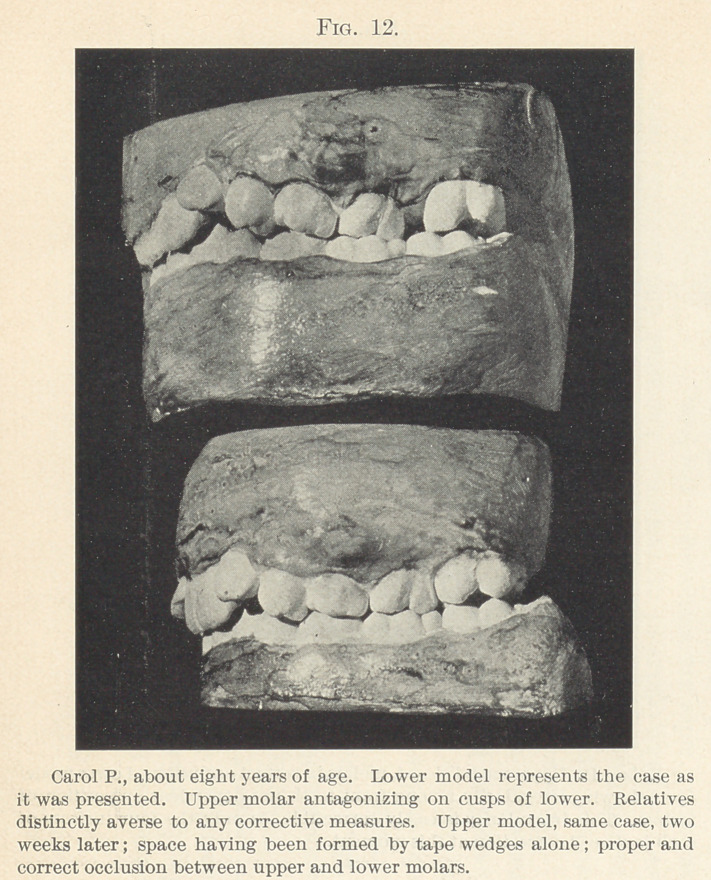


**Fig. 13. f14:**
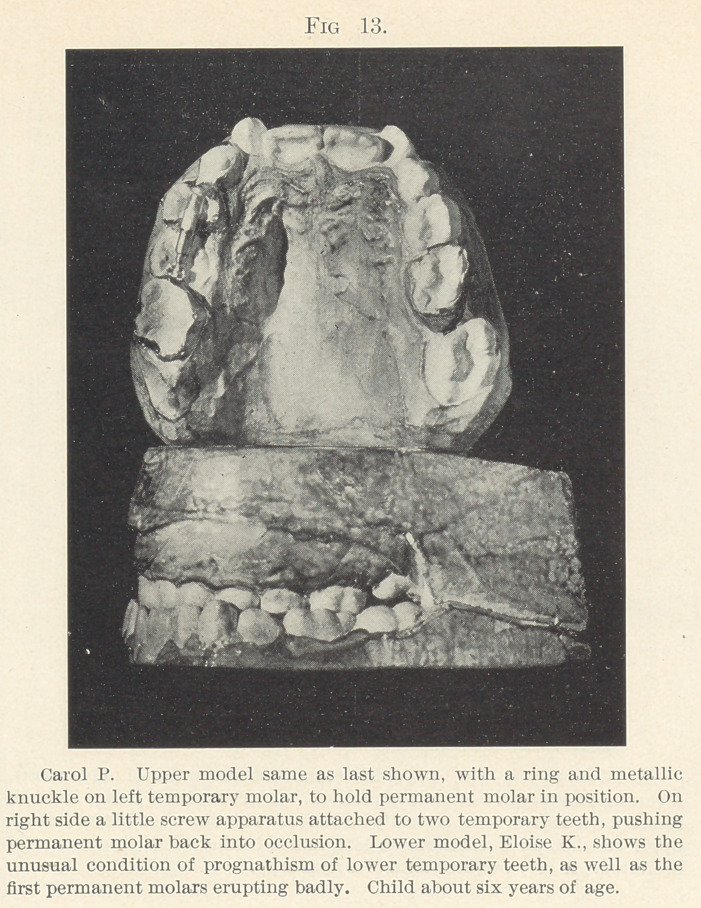


**Fig. 14. f15:**
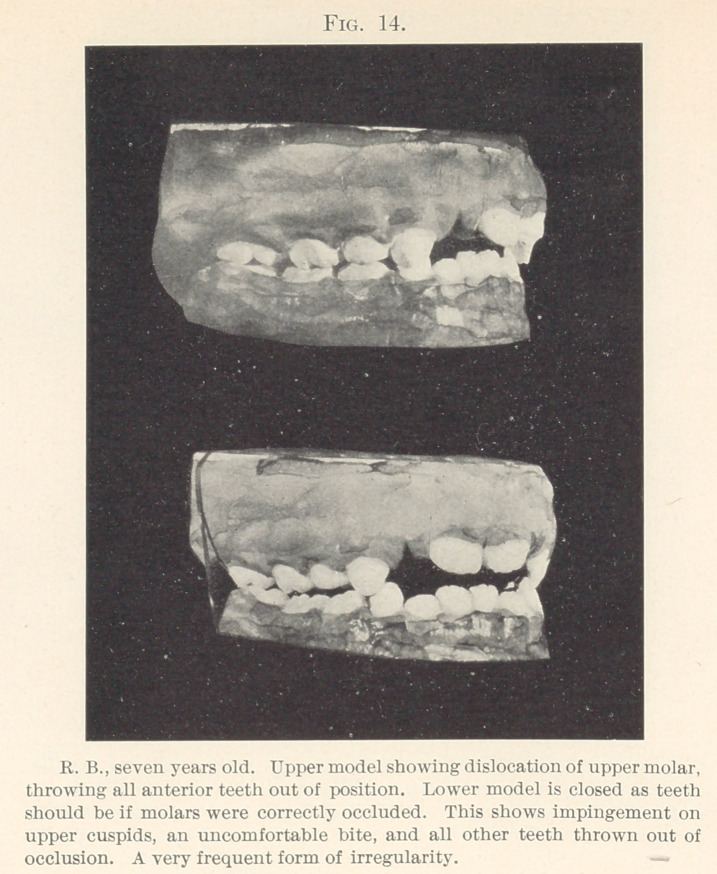


**Fig. 15. f16:**
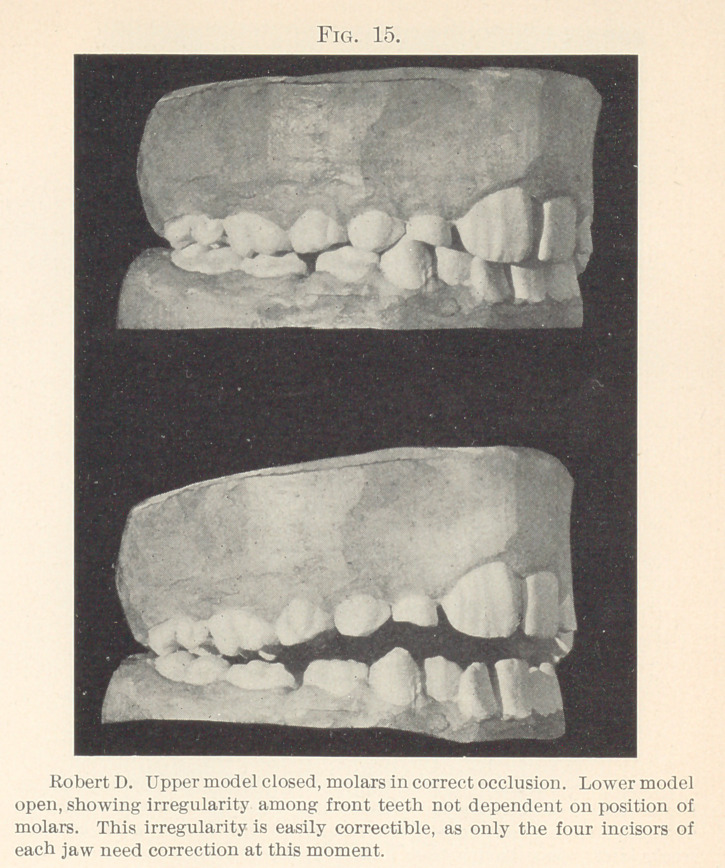


**Fig. 16. f17:**
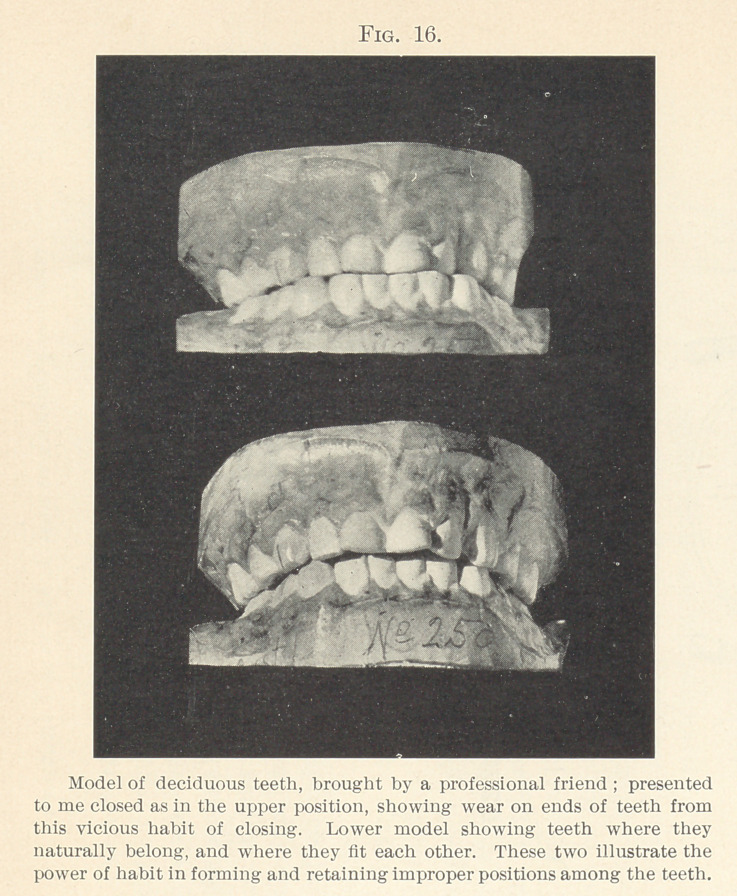


**Fig. 17. f18:**
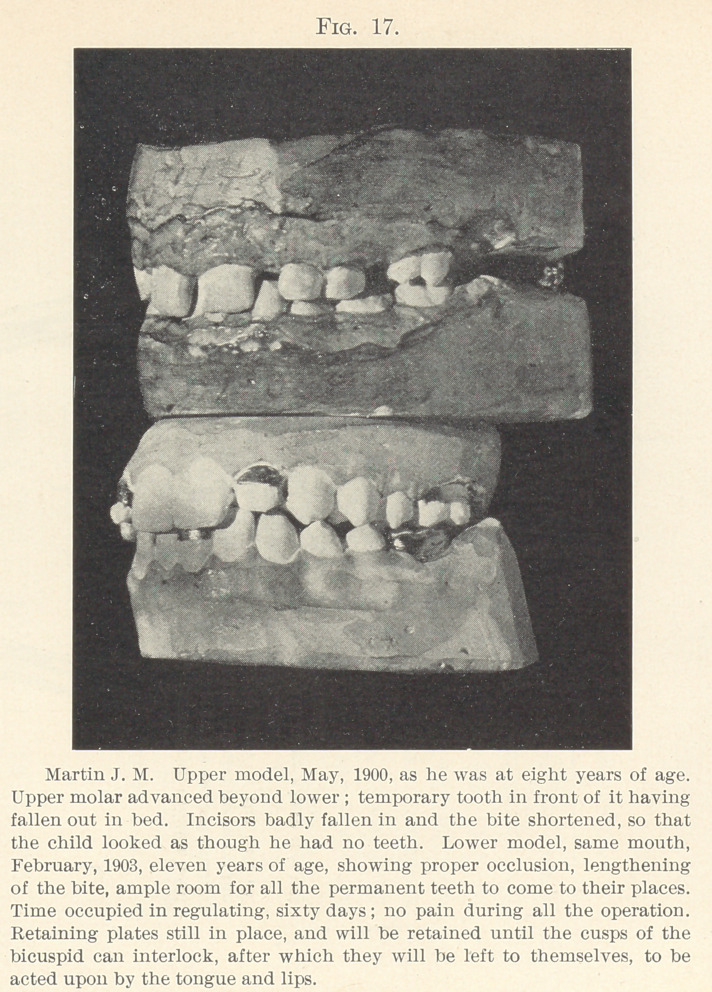


**Fig. 18. f19:**